# Prevalence of diabetes mellitus in patients with acromegaly

**DOI:** 10.1530/EC-14-0021

**Published:** 2014-04-29

**Authors:** A V Dreval, I V Trigolosova, I V Misnikova, Y A Kovalyova, R S Tishenina, I A Barsukov, A V Vinogradova, B H R Wolffenbuttel

**Affiliations:** Moscow Regional Scientific Research Clinical Institute61/2 Shepkina str., 129110 Moscow, Russia ^1^ Department of EndocrinologyUniversity of Groningen, University Medical Center Groningen, PO Box 30001, 9700 RB Groningen, The Netherlands

**Keywords:** acromegaly, glucose, diabetes, IGF1, epidemiology

## Abstract

Early carbohydrate metabolism disorders (ECMDs) and diabetes mellitus (DM) are frequently associated with acromegaly. We aimed to assess the prevalence of ECMDs in patients with acromegaly and to compare the results with those in adults without acromegaly using two population-based epidemiologic surveys. We evaluated 97 patients with acromegaly in several phases of their disease (mean age, 56 years and estimated duration of acromegaly, 12.5 years). An oral glucose tolerance test was done in those not yet diagnosed with DM to reveal asymptomatic DM or ECMDs (impaired glucose tolerance+impaired fasting glucose). Comparisons were made between patients with acromegaly and participants from the general adult population (*n*=435) and an adult population with multiple type 2 diabetes risk factors (*n*=314), matched for gender, age and BMI. DM was diagnosed in 51 patients with acromegaly (52.5%) and 14.3% of the general population (*P*<0.001). The prevalence of ECMDs was also higher in patients with acromegaly than in the general population and in the high-risk group; only 22% of patients with acromegaly were normoglycaemic. The prevalence of newly diagnosed ECMDs or DM was 1.3–1.5 times higher in patients with acromegaly compared with the high-risk group. Patients with acromegaly having ECMDs or DM were older, more obese and had longer disease duration and higher IGF1 levels (*Z*-score). Logistic regression showed that the severity of glucose derangement was predicted by age, BMI and IGF1 levels. In patients with acromegaly, the prevalence of DM and ECMDs considerably exceeds that of the general population and of a high-risk group, and development of DM depends on age, BMI and IGF1 levels.

## Introduction

Disturbances of glucose metabolism are frequently observed in patients with acromegaly. In one of the first papers to be published on this topic, abnormal glucose tolerance was found in over 60% of patients with acromegaly (1). The glucose anomalies in these patients are now known to include diabetes mellitus (DM), impaired glucose tolerance (IGT) and impaired fasting glucose (IFG) and have been discussed extensively in a review by Colao *et al*. (2). Looking more specifically at early carbohydrate metabolism disorders (ECMDs) – defined as IFG, IGT or their combination – its prevalence in patients with acromegaly has been shown to vary between 16 and 46% (3, 4, 5). While most epidemiological studies have shown the prevalence of ECMDs to be higher than that of overt diabetes (2, 5, 6, 7), not all studies report the same prevalence (3, 8). The development of ECMDs and/or progression to diabetes in patients with acromegaly may depend on several factors, such as age and gender (5), the levels of growth hormone (GH) (9), as well as the duration of acromegaly and duration of exposure to elevated GH levels (9, 10). However, other authors have found no differences in GH levels and insulin-like growth factor 1 (IGF1) levels or disease duration between those with glucose disturbances and those who were normoglycaemic (5, 6, 10, 11). A further possible factor involved in the early development of diabetes is a positive family history of DM (5, 12). A final factor that may also influence the development of glucose disturbances is the specific treatment for acromegaly. Somatostatin analogues may influence glucose metabolism both by lowering insulin secretion and by lowering GH and IGF1 levels (13, 14).

The aim of this study was to assess the prevalence of ECMDs and DM in patients with acromegaly undergoing treatment at a large tertiary referral centre in Moscow (15) and to compare the results with the prevalence of such glucose disturbances in adults without acromegaly using two population-based surveys (16, 17). We also analysed the factors contributing to the development of hyperglycaemia.

## Subjects and methods

### Patients

A total of 97 patients with acromegaly undergoing treatment or long-term follow-up at the outpatient clinic of the Moscow Regional Clinical Research State Institute underwent an extensive evaluation, which included measurement of height, weight and blood pressure. Those patients who had not previously been diagnosed with DM underwent an oral glucose tolerance test (OGTT). In the fasting state, and 30, 60, 90 and 120 min after oral administration of 75 g glucose, blood was drawn for the simultaneous measurement of plasma levels of GH and glucose. In patients, who had already been diagnosed with DM, GH levels were measured in the fasting state and subsequently every 30 min for 2 h. As hypopituitarism or treatment for hypopituitarism can influence carbohydrate metabolism, we did not include patients requiring such therapy.

The prevalence of disturbed glucose metabolism was compared between patients with acromegaly and adults without acromegaly using the results of two population-based studies, during which study an OGTT was performed. The first study was performed in 2006 among a random sample of the adult population living in two districts of the Moscow region, i.e. the Lukhovitsky district in the southeastern part of the Moscow Region (58 800 inhabitants) and the town of Zhukovsky (105 332 inhabitants). The second study was performed between 2009 and 2010 among an adult population, who had a high risk of developing type 2 diabetes (T2DM), living in three different districts (Kashirsky, Mozhaisky and Kolomensky districts) established with the FINDRISK questionnaire. A risk score of 12 was selected as the cut-off point for categorising a person at high risk for DM (18). For each patient with acromegaly, up to five participants from both screening studies were matched for age, gender and BMI.

ECMDs (IGT and IFG) and T2DM were diagnosed according to the WHO recommendations (19). The activity of acromegaly was assessed as described in the 2009 international consensus statement (20). The duration of acromegaly was estimated starting from when the first complaints of specific changes in physical appearance occurred.

The studies were approved by the Medical Ethical Review Committee of the Moscow Regional Clinical Research State Institute.

### Laboratory measurements

Serum concentrations of GH were measured by a two-site IRMA (Immunotech SA, Prague, Czech Republic; intra-assay variation, 0.6% and inter-assay variation, 13.5%), and levels of IGF1 were measured by an IRMA (Immunotech SA; intra-assay variation, 7.1% and inter-assay variation, 11.9%) using a Beckman Coulter device (Beckman Coulter, Villepinte, France). Plasma glucose levels were measured using a hexokinase method on a Hitachi 912 analyzer (Hoffmann-La Roche Ltd/Roche Diagnostics GmbH).

### Statistical analyses

All statistical analyses were performed using IBM SPSS Statistics, version 20 for Windows (IBM, Armonk, NY, USA). Baseline characteristics were reported as mean and interquartile range (IQR) for continuous variables and as number and percentage for dichotomous variables. To compare unpaired variables, the *χ*^2^-test or Mann–Whitney *U* test was used. The differences were considered statistically significant at *P*<0.05. Logistic regression analysis using a multinomial logit model was used to identify risk factors for diabetes or ECMDs. The independent variables used in this analysis were age, BMI (continuous), duration of acromegaly, fasting GH, IGF1, treatment with an somatostatin analogue (SSA) (yes/no) and previous transsphenoidal pituitary surgery (yes/no).

## Results

The characteristics of patients with acromegaly are given in Tables 1 and 2. The mean age of patients with acromegaly was 56 years (IQR 47–64). Of the 97 patients, 81 were women, and the mean estimated duration of acromegaly was 13 years (IQR 7–20). Patients were in different phases of their disease: 16 patients had newly diagnosed acromegaly, 38 subjects had previously undergone transsphenoidal surgery (TSS) and 65 were being treated with an SSA, either directly after diagnosis (*n*=40), or because of persistent GH overproduction despite TSS (*n*=25). GH overproduction was controlled in 28 of the 97 individuals. None of the patients were treated with a dopamine agonist or with pegvisomant.

**Table 1:** Comparative characteristics of patients with acromegaly and matched participants from the general population.

	**Patients with acromegaly**	**General population**
Number of patients/participants	97	435
Male/female (%)	16/84	13/87
Age (years)	56 (47.5–64.5)	56 (47–64)
BMI (kg/m^2^)	31.0 (27.7–34.2)	30.6 (27.2–34.1)
SBP (mmHg)	**138±20**	**140±24**
DBP (mmHg)	**86±13**	**86±12**
Known diabetes, *n* (%)	24 (24.7)	30 (6.9)
Newly diagnosed diabetes, *n* (%)	27 (27.8)	32 (7.4)
Total diabetes, *n* (%)	51 (52.5)	62 (14.3)
Isolated IFG, *n* (%)	10 (10.3)	45 (10.3)
Isolated IGT, *n* (%)	4 (4.1)	14 (3.2)
Combination of IFG+IGT, *n* (%)	11 (11.3)	23 (5.3)
ECMDs, *n* (%)	25 (25.8)	82 (18.8)
Normoglycaemia, *n* (%)	21 (21.6)	291 (66.9)

Data are given as absolute numbers (*n*) and percentage (%) or median (interquartile range). Groups were matched according to gender, age and BMI. SBP, systolic blood pressure; DBP, diastolic blood pressure; IFG, impaired fasting glucose; IGT, impaired glucose tolerance; ECMDs, early carbohydrate metabolism disorders.

**Table 2:** Comparative characteristics and results of the oral glucose tolerance test in subjects not previously known to have diabetes.

	**Patients with acromegaly**	**General population**	**High-risk group**
Number of patients/participants	73	325	314
Males/females (%)	19/81	15/85	15/85
Age (years)	54 (47–60)	54 (46–62)	54 (48–62)
BMI (kg/m^2^)	30.1 (26.9–34.0)	29.6 (26.5–33.9)	30.5 (27.2–35.1)
SBP (mmHg)	**135±19**	**137±24**	**143±23**
DBP (mmHg)	**84±11**	**85±12**	**88±12**
Newly diagnosed diabetes, *n* (%)	27 (37.0)	31 (9.5)	77 (24.5)
Isolated IFG, *n* (%)	10 (13.7)	36 (11.1)	32 (10.2)
Isolated IGT, *n* (%)	4 (5.5)	13 (4.0)	32 (10.2)
Combination of IFG+IGT, *n* (%)	11 (15.1)	11 (3.4)	21 (6.7)
ECMDs, *n* (%)	25 (34.2)	60 (18.5)	85 (27.1)
Normoglycaemia, *n* (%)	21 (28.8)	234 (72.0)	152 (48.4)

Data are given as absolute numbers (*n*) and percentage (%) or median (interquartile range). Groups were matched for gender, age and BMI. SBP, systolic blood pressure; DBP, diastolic blood pressure; IFG, impaired fasting glucose; IGT, impaired glucose tolerance; ECMDs, early carbohydrate metabolism disorders.

In 24 patients with acromegaly, DM had previously been diagnosed during the course of their disease. Out of 24, six were being treated with a diet only, six with oral blood glucose lowering agents (either metformin or sulphonylurea), two with insulin alone and ten with insulin plus an oral agent (metformin or sulphonylurea). In 27 of the 73 patients who underwent an OGTT, diabetes was diagnosed, resulting in a total number of 51 (52.5%) patients with DM. This prevalence was 3.5 times higher than the prevalence of T2DM in the general population (14.3%, Table 1). When we analysed the results of the OGTT performed in those not known to have diabetes, we observed a higher prevalence of newly diagnosed diabetes and ECMDs in patients with acromegaly compared with both population-based screening groups (Table 2).

**Figure 1:**
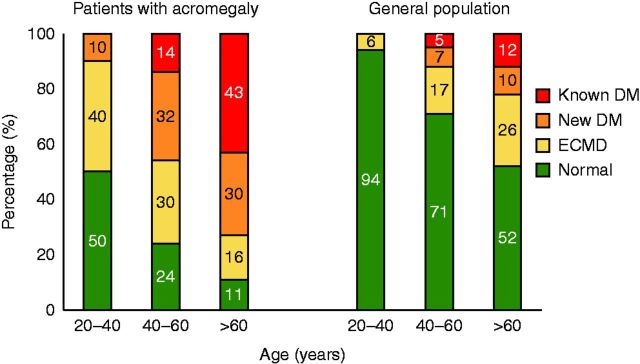
Prevalence of carbohydrate metabolism disturbance according to age in patients with acromegaly and the general adult population.

In Fig. 1, we show that there is a gradual increase in diabetes prevalence with age in the general population and in patients with acromegaly. In patients with acromegaly, DM as well as all disturbances in glucose metabolism is more prevalent when compared with the general population in all age groups. Only 28.8% of patients with acromegaly were proved to have normal glucose tolerance (NGT).

Table 3 lists the differences in clinical and biochemical characteristics in patients with acromegaly according to disturbances in glucose metabolism. Subjects with ECMDs and diabetes were older and more obese, and the diabetes group had the highest proportion of women. The duration of acromegaly was longest in the diabetes group, and IGF1 levels were higher in subjects with ECMDs and diabetes than in individuals with NGT. When we looked at the different age groups, we found the duration of clinical symptoms to be longer in the oldest subjects (those >60 years) than in subjects in the age group of 20–40 years (median 16.7 vs 8.0 years, *P*<0.01).

**Table 3:** Characteristics of the different groups of patients with acromegaly according to disturbances of glucose metabolism.

**Parameters**	**NGT**	**ECMDs**	**DM**	**P value**
Number of patients	21	25	51	–
Male/female, *n*	7/14	4/21	5/46	0.050
Age (years)	47±12	53±12	60±11	<0.001
BMI (kg/m^2^)	28.4±2.8	31.2±5.4	33.1±5.8	0.009
SBP (mmHg)	**131±21**	**132±16**	**144±21**	**0.014**
DBP (mmHg)	**83±12**	**82±11**	**89±13**	**0.061**
Estimated duration of acromegaly (years)	9.0 (5.4–12.8)	11.0 (6.8–20.0)	15.0 (8.0–24.0)	0.013
Basal GH (mU/l)	4.2 (1.1–8.4)	6.2 (3.1–16.4)	5.7 (2.2–15.6)	0.609
IGF1 (ng/ml)	223 (147–436)	403 (252–663)	310 (194–582)	0.460
IGF1 *Z*-score	2.1 (0.7–4.4)	5.1 (3.3–6.6)	4.5 (2.3–6.5)	0.002
Previous TSS, *n* (%)	14 (67)	10 (40)	14 (27)	0.008
Use of SSA, *n* (%)	11 (54)	19 (76)	35 (69)	0.222
Acromegaly controlled, *n* (%)	**8 (38)**	**3 (12)**	**11 (22)**	**0.105**

Data are given as absolute numbers (*n*) and percentage (%), mean±s.d. or median (interquartile range). SBP, systolic blood pressure; DBP, diastolic blood pressure; GH, growth hormone; TSS, transsphenoidal surgery; SSA, somatostatin analogue; NGT, normal glucose tolerance; ECMDs, early carbohydrate metabolism disorders; DM, diabetes mellitus.

Logistic regression analysis showed that the presence of glucose derangement was predicted by age, BMI and levels of IGF1. The odds of an acromegaly patient progressing to ECMDs or diabetes for each of these parameters are depicted in Table 4.

**Table 4:** Odds ratios for the presence of ECMDs and DM in patients with acromegaly.

**Parameters**	**NGT**	**ECMDs**	**DM**	**ECMDs+DM**
Age (per s.d.)	1.0	1.10 (0.43–2.82)	2.74 (1.11–6.8)^*^	1.98 (0.86–4.5)
BMI (per s.d.)	1.0	1.73 (0.58–5.2)	3.26 (1.14–9.3)^*^	2.70 (0.98–7.4)
IGF1 *Z*-score	1.0	1.58 (1.09–2.27)^*^	1.50 (1.06–2.11)^*^	1.48 (1.07–2.06)^*^

Data are given as ORs with 95% CI, adjusted for age, BMI, duration of acromegaly, fasting growth hormone levels, IGF1 *Z*-score and treatment with somatostatin analogues. NGT, normal glucose tolerance (reference group); ECMDs, early carbohydrate metabolism disorders; DM, diabetes mellitus. *P<0.05 vs NGT.

## Discussion

In this paper, we have described a high prevalence of DM and ECMDs in patients with acromegaly. Over 50% of our patients had DM, either overt and already being treated with diet or medication or diagnosed with an OGTT during our study. In a further 26% of subjects, the OGTT revealed the presence of IFG or IGT, together named ECMDs. The prevalence of both DM and ECMDs was significantly higher in patients with acromegaly than in the general population or in a population with a high risk of diabetes; only 22% of subjects with acromegaly were normoglycaemic. This report on glucose metabolism derangements in a large population of patients with acromegaly in Russia confirms earlier reports demonstrating the prevalence of ECMDs to be higher in these patients than in the general population (2, 5, 6, 7).

In accordance with these earlier observations, we observed those patients who had already developed diabetes or ECMDs to be older and have a higher BMI, and a longer duration of acromegaly. In addition, our logistic regression models showed that age, BMI and levels of IGF1 were independently associated with the presence of ECMDs and DM. Our finding that levels of IGF1, but not levels of GH, could predict abnormal glucose metabolism is partly in accordance with the observations of others. These two risk factors have been assessed in several studies (2, 5, 6, 7), but the results were conflicting. A Polish study in 220 treatment-naïve patients with acromegaly reported no significant differences in basal plasma GH, IGF1 or fasting insulin concentrations between normoglycaemic patients and those with impairments in glucose tolerance (6), although the latter group of patients was significantly older. With regard to IGF1, a recent paper reported that higher IGF1 levels were associated with more severe insulin resistance and hyperglycaemia (12). In accordance with the data of Colao *et al*. (14), the presence of DM was associated with a higher age and longer disease duration, and in addition IGF1 levels predicted fasting as well as 2 h glucose levels and HbA1c after 12 months of SSA treatment. The effects of GH and IGF1 on glucose metabolism are complex. On the one hand, GH hypersecretion leads to an increase in insulin resistance (decrease in glucose uptake and glycolysis and increase in availability of free fatty acids) in adipose tissue and muscles, and to an increase in gluconeogenesis in hepatocytes (21), and indeed, a significant correlation between plasma GH and IGF1 concentrations and measures of insulin resistance has been reported (6). On the other hand, IGF1 has a positive effect on hybrid insulin/IGF1 receptors in adipose tissue, muscles and hepatocytes, which ensures its insulin-like action (21). IGF1-dependent gastric inhibitory polypeptide (GIP) secretion additionally stimulates insulin secretion (22). IGF1 also increases the sensitivity of hepatocytes to the effects of GH (21, 23), which are diabetogenic as mentioned above. An additional antidiabetogenic effect may be exerted by somatostatin analogues, which decrease GH hypersecretion, and long-acting octreotide in particular has been associated with a better control of IGF1 (24). Nevertheless, these medications appear to decrease insulin secretion.

A final important finding was that patients who had undergone TSS had a lower prevalence of glucose derangements: the percentage of patients with NGT was three times higher in the group that had undergone TSS. However, in regression analysis, previous TSS was not an independent predictor of glucose derangements, probably because those who had undergone TSS had a mean age 8 years younger than those who had not undergone surgery.

We conclude that disturbances of glucose metabolism frequently develop in patients with acromegaly. The prevalence of DM and ECMDs among patients with acromegaly considerably exceeds the prevalence of T2DM and ECMDs in the general population and in a high-risk population. Its development depends on age, BMI and IGF1 levels.

## Declaration of interest

The authors declare that there is no conflict of interest that could be perceived as prejudicing the impartiality of the research reported.

## Funding

The studies described in this paper were supported financially by the Ministry of Health of the Russian Federation.
